# Enhancement of Biomass Conservation and Bioethanol Production of Sweet Sorghum Silage by Constructing Synergistic Microbial Consortia

**DOI:** 10.1128/spectrum.03659-22

**Published:** 2023-01-16

**Authors:** Yu-Xi Zhu, Xu Zhang, Wen-Chao Yang, Jun-Feng Li

**Affiliations:** a College of Plant Protection, Yangzhou University, Yangzhou, China; b College of Agro-grassland Science, Nanjing Agricultural University, Nanjing, China; c College of Agronomy and Horticulture, Jiangsu Vocational College of Agriculture and Forestry, Nanjing, China; Migal-Galilee Research Institute

**Keywords:** microbes, sweet sorghum, biomass preservation, ensiling

## Abstract

The efficient storage of materials before bioethanol production could be key to improving pretreatment protocol and facilitating biodegradation, in turn improving the cost-effectiveness of biomass utilization. Biological inoculants were investigated for their effects on ensiling performance, biodegradability of silage materials, and final bioethanol yield from sweet sorghum. Two cellulolytic microbial consortia (CF and PY) were used to inoculate silages of sweet sorghum, with and without combined lactic acid bacteria (Xa), for up to 60 days of ensiling. We found that the consortia notably decreased pH and ammonia nitrogen content while increasing lactic acid/acetic acid ratios. The microbes also functioned in synergy with Xa, significantly reducing lignocellulose content and improving biomass preservation. First-order exponential decay models captured the kinetics of nonstructural carbohydrates and suggested high water-soluble carbohydrate (grams per kilogram dry matter [DM]) preservation potential in PY-Xa (33.48), followed by CF-Xa (30.51). Combined addition efficiently improved enzymatic hydrolysis and enhanced bioethanol yield, and sweet sorghum treated with PY-Xa had the highest ethanol yield (28.42 g L^−1^). Thus, combined bioaugmentation of synergistic microbes provides an effective method of improving biomass preservation and bioethanol production from sweet sorghum silages.

**IMPORTANCE** Ensiling is an effective storage approach to ensure stable year-round supply for downstream biofuel production; it offers combined facilities of storage and pretreatment. There are challenges in ensiling sweet sorghum due to its coarse structure and high fiber content. This study provides a meaningful evaluation of the effects of adding microbial consortia, with and without lactic acid bacteria, on changes in key properties of sweet sorghum. This study highlighted the bioaugmented ensiling using cellulolytic synergistic microbes to outline a cost-effective strategy to store and pretreat sweet sorghum for bioethanol production.

## INTRODUCTION

Biofuels are essential to the transition from fossil fuels toward cleaner renewable energy (1). Lignocellulosic biomasses, which can be converted into second-generation bioethanol, are a potential source of this clean energy (2). However, the occurrence of biomass recalcitrance has continued to hinder its application. To facilitate the production and use of bioethanol at industrial scales, thorough consideration of the economic, environmental, and sustainability aspects related to bioconversion processes that turn lignocellulosic biomasses into bioethanols is required. Further, bioethanol production is tightly linked to feedstock availability, infrastructure support such as supply chains and technologies, and the cost-effectiveness of the process (3).

Sweet sorghum (Sorghum bicolor L. Moench), a known C4 energy crop, can provide grains and sugars while also offering lignocellulosic resources (4). This gives it enormous potential to be used for bioethanol production. Sweet sorghum has high cellulosic fiber content (27 to 44.6%), high productivity, wide adaptability, and low agricultural inputs, resulting in it being highly suitable for industrial cellulosic-ethanol production (5). Despite it being deemed a potential candidate biofuel feedstock, the seasonality of sweet sorghum greatly hinders its sustainability and economic viability. Another drawback of the crop is that it is highly susceptible to microbial contamination. After harvest, crops are frequently affected by mildew, which leads to rapid deterioration and the loss of soluble sugars, which, in turn, lowers biofuel yield. To prevent this, ensiling is an effective storage approach that provides both storage and pretreatment to prevent contamination and secure a year-round supply of material for downstream production (6, 7). Using lactic acid bacteria to change accessible substrates to organic acids, ensiling results in biological acidification, stabilizing the biomass, and promoting preservation (8). Unsurprisingly, many studies target biomass silage as a material supply for biofuels or biogases production (9, 10). Beyond this, efficient storage can serve as the upstream process that enhances enzymatic hydrolysis or ethanol fermentation. Enabling such processes could significantly extend the efficacy of pretreatments, hence improving the output or reducing the cost of biomass utilization. Nevertheless, it is a challenge to ensile sweet sorghum because of the high content of fiber in sorghum. Cellulose, the main carbon substrate in lignocellulosic biomass, is considered the crucial carbon reservoir to transform into glucose residues (11). Therefore, improving the transformation of cellulosic biomass is necessary to achieve the efficient utilization of sweet sorghum.

The addition of microorganisms in sweet sorghum silage, specifically, those encoding endoglucanase, exoglucanase, β-glucosidase, or other enzyme families of glycosyl hydrolases (GHs) can facilitate biomass depolymerization (12). This, in turn, accelerates processes such as hydrolysis, production of soluble carbohydrates, and fermentation by lactic acid bacteria. Microbes known to degrade lignocelluloses do so in a synergistic and complex process that involves several different secreted enzymes (13). Compared to individual strains, microbial consortia that contain many different genera often perform better and faster biodegradation of lignocellulosic biomass due to synergistic metabolic activities. Srivastava et al. (14) found that consortium SNH-1 from camel rumen prominently enhanced hydrolysis of wheat straw compared with the single microorganism. Ali et al. (15) found that using synergistic microbial consortia significantly improved biogas and methane yields from catalpa sawdust. One frequent source of these microbes or metabolic enzymes is rumen of ruminants. Rumen bacteria often exhibit higher ability and activity for lignocellulose degradation than other anaerobic microorganisms (16). Moreover, their high growth rate, high metabolic activity, and survival under industrial environments make them top candidates for biofuel production. Application of these rumen bacteria as inoculum could be crucial to improving the efficiency and scale of bioethanol production using lignocellulosic biomasses.

In our previous work, we extracted novel consortia from the rumen of the Tibetan yak and found that they could be used to ensile rice straw, which greatly improved enzymatic hydrolysis as well as ethanol production. However, the results are not necessarily directly applicable to other crops, as every type of lignocellulosic substrate varies in composition and thus requires tailored pretreatments to maximize biofuel production (17). Epiphytic microorganisms further complicate the situation for fresh materials, as their composition, and thus, a response to inoculants, can vary with plant sources. The synergistic action of the consortia could improve substrate utilization and enable production under nonsterile conditions, thus reducing production costs. The aim of the study was to investigate whether the addition of cellulolytic microbial consortia could improve biomass preservation and bioethanol production from sweet sorghum silages. We examined the effects of adding microbial consortia, with and without lactic acid bacteria, on changes in key properties of sweet sorghum, including composition, structure, fermentation dynamics, as well as its overall efficiency when used in ethanol production.

## RESULTS AND DISCUSSION

As we previously described, *Enterococcus*, Klebsiella, and Escherichia were the dominant genera in the two consortia (CF and PY) (see Table S1 in the supplemental material) (18). These predominant bacteria genera from the two consortia (CF and PY) produce a diverse array of CAZyme family genes (Table S2), which are instrumental to their strong cellulose-metabolizing capabilities (18). Specifically, several major GH families (e.g., cellulases, hemicellulases, etc.), known to be abundant in other lignocellulolytic microbiota, are also present here (19, 20). Metagenomic analysis of CAZymes during the degradation of rice straw showed that GHs were the main family of functional enzymes involved (19–21). GHs are known to depolymerize complex polysaccharides by targeting glycosidic bonds, creating fermentable sugars. Based on the higher number of such genes in PY, it is also likely that these consortia have a stronger cellulose degradation ability than CF.

### Effects of initial medium pH on the activity of multiple enzymes.

**FIG 1:**
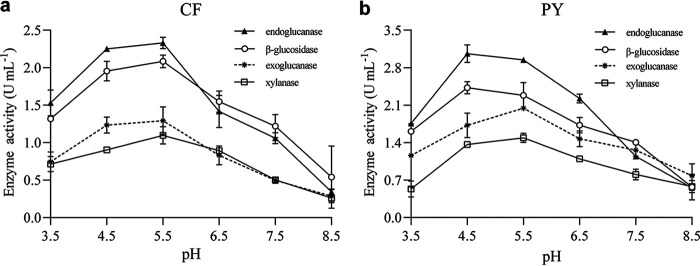
Influence of pH on enzyme activities by consortium CF (a) and consortium PY (b) (*n* = 5; standard error indicated by bars).

Nutrient transport and enzymatic systems were expected to be significantly affected by initial pH levels (18). This influence then extends to cell growth and enzyme production. Therefore, enzymatic activities of CF and PY were tested at different pH levels, simulating the range of the pH before ensiling and after an effective fermentation. The synergistic interaction between cellulases and xylanase can reduce the “blocking effect” known of xylan, thus greatly increasing cellulose accessibility. Therefore, we also evaluated how initial pH levels would affect the characteristics of these enzymes (Fig. 1). At an initial pH of 4.5, activities of endoglucanase and β-glucosidase from consortium PY reached their maximum of 3.06 and 2.43 U mL^−1^, respectively. At higher pH levels (>5.5), the endoglucanase and β-glucosidase activities of consortium CF reached the maximum value (2.33 and 2.09 U mL^−1^, respectively). As for exoglucanases and xylanases, activity levels peaked when the initial pH was around 5.5: in those produced by consortium CF, activity levels were 1.29 and 1.10 U mL^−1^, respectively, whereas those produced by PY reached 2.04 and 1.49 U mL^−1^ in activity levels. Compared to alkaline conditions (pH 7.5 to 8.5), slightly acidic conditions were beneficial for enzyme production in the consortia CF and PY. Nonetheless, the lignocellulolytic enzymes produced by cellulolytic microbial consortia were acid resistant and remained active over a wide range of pH conditions, which was expected based on the observations (22, 23). Considering these characteristics, our results support that the microbial consortia CF and PY possess enormous potential for silage making.

### Fermentation profile of sweet sorghum silages.

The dynamics of fermentation characteristics of sweet sorghum silage were as documented in Tables 1 and 2. The type of treatment, as well as the number of ensiling days, significantly affected the pH value, dry matter (DM), lactic acid (LA), acetic acid (AA), ratio of lactic acid to acetic acid (LA/AA), and NH_3_-N content (in all cases, *P < *0.05). As previously reported, the initial stages of ensiling had adequate levels of water-soluble carbohydrate (WSC), which leads to accelerated homofermentation by either epiphytic or inoculated lactic acid bacteria and results in a rapid increase in LA content in the silages (8, 24). The pH values decreased most notably and continued to drop below 4.2 on day 7. In the CF and PY treatments, this pH persisted until the end of ensiling. Importantly, this pH satisfies the 3.6 to 4.2 range that indicates well-preserved silages and thus reflects fermentation quality by these two consortia (25). Compared to the control, treatment with each consortia individually or combined with Xa treatment all significantly increased LA content, improved LA/AA content, and reduced volatile fatty acid and NH_3_-N values. PY-Xa and CF-Xa had significantly higher LA/AA values and lower pH values and NH_3_-N than those inoculated with CF, PY, or Xa alone (*P < *0.05). The highest LA content (85.91 g kg^−1^ DM) and lowest pH value (3.81) were recorded in the PY-Xa silage. These outcomes suggested that the cellulolytic microbial consortia provide the soluble saccharides, while lactic acid bacteria utilize them for accelerating LA fermentation and improving silage quality. A similar bioprocess that directly produces lactic acid from lignocellulosic biomasses by applying an artificially synthesized consortium of cellulolytic fungus and lactic acid bacteria had also been established by Shahab et al. (26). Also, Xu et al. (27) found that the growth of lactic acid bacteria cocultured with lignocellulosic biomass-degrading Paenibacillus panacisoli was better than lactic acid bacteria alone, and many organic acids were released by cocultivation in corn stover silages.

**TABLE 1:** Influence of microbial consortia on dry matter, pH value, lactic acid, and ammonia nitrogen contents in sorghum silage during ensiling^*a*^

Item	Treatment	Data for ensiling day:					SEM	*P* value		
		3	7	14	30	60		*T*	*D*	*T*×*D*
DM (g kg^−1^ FW)	CK	205.71^A^	195.67^AB^	182.98^BC^	172.37^C^	146.16^bD^	6.808	0.001	<0.001	0.978
	Xa	218.18^A^	192.85^B^	180.55^BC^	173.94^C^	150.77^bD^				
	CF	215.71^A^	201.35^A^	183.38^B^	176.96^BC^	165.14^abC^				
	PY	211.51^A^	192.85^AB^	183.88^BC^	173.94^BC^	164.10^abC^				
	CF-Xa	221.07^A^	205.48^AB^	191.94^BC^	185.44^BC^	178.30^aC^				
	PY-Xa	221.76^A^	206.11^AB^	193.98^AB^	185.94^B^	177.77^aB^				
pH	CK	4.63^aB^	4.48^aBC^	4.37^aC^	4.39^aC^	4.46^aA^	0.026	<0.001	0.051	0.678
	XA	4.13^b^	3.98^b^	3.99^b^	4.24^ab^	4.30^ab^				
	CF	4.29^b^	4.14^b^	4.14^b^	4.18^ab^	4.08^b^				
	PY	4.18^b^	4.05^b^	4.01^b^	4.06^ab^	4.14^b^				
	CF-Xa	4.19^b^	4.05^b^	3.95^b^	3.88^b^	3.85^c^				
	PY-Xa	4.14^b^	4.10^b^	4.01^b^	3.94^ab^	3.81^c^				
Lactic acid (g kg^−1^ DM)	CK	37.78^bCD^	45.64^bB^	49.98^bAB^	50.96^cA^	30.36^cD^	1.414	<0.001	<0.001	<0.001
	Xa	57.29^aAB^	70.93^aA^	70.81^aA^	62.27^bAB^	53.96^bB^				
	CF	52.50^aC^	63.84^aB^	64.26^aB^	64.84^bB^	72.60^abA^				
	PY	63.46^aB^	69.38^aAB^	72.58^aAB^	74.41^abAB^	75.65^abA^				
	CF-Xa	61.55^aD^	68.51^aC^	75.56^aB^	83.18^aA^	81.30^aAB^				
	PY-Xa	63.96^aC^	66.87^aC^	71.48^aBC^	79.74^aAB^	85.91^aA^				
Ammonia nitrogen (g kg^−1^ TN)	CK	46.29^aD^	55.16^aCD^	66.05^aBC^	76.21^aB^	79.93^aA^	1.389	<0.001	<0.001	0.982
	Xa	34.90^abB^	39.47^bB^	40.72^bcB^	47.26^bAB^	50.37^bcA^				
	CF	31.13^bC^	39.56^bBC^	45.03^bB^	58.28^bAB^	56.96^bA^				
	PY	32.88^bC^	34.54^bBC^	45.24^bBC^	55.80^bAB^	58.79^bA^				
	CF-Xa	30.12^bB^	31.39^bAB^	35.64^bAB^	38.32^bA^	48.08^cA^				
	PY-Xa	30.82^bB^	37.81^bAB^	34.08^bAB^	41.32^bAB^	49.08^cA^				

a Values with different small letters indicate significant differences among treatments over the same ensiling days, and values with different capital letters show significant differences among ensiling days in the same treatment (*P* < 0.05). *T*, treatment; *D*, ensilage time; *T*×*D*, interaction between treatment and ensilage time; DM, dry matter; FW, fresh material; TN, total nitrogen; CK, control; Xa, only inoculated with combined lactic acid bacteria; CF, only adding consortium CF; PY, adding consortium PY; CF-Xa, adding both consortium CF and Xa; PY-Xa, adding both consortium PY and Xa.

**TABLE 2:** Influence of microbial consortia on the content of volatile fatty acid and lactic acid/acetic acid in sorghum silage during ensiling^*a*^

Item	Treatment	Data for ensiling day:					SEM	*P* value		
		3	7	14	30	60		*T*	*D*	*T*×*D*
Acetic acid (g kg^−1^ DM)	CK	25.16^aC^	27.21^aC^	30.68^aBC^	33.79^aB^	39.97^aA^	0.633	<0.001	<0.001	0.127
	Xa	15.74^bC^	19.46^bBC^	21.31^bAB^	22.56^bAB^	25.15^bA^				
	CF	17.70^bB^	20.28^bAB^	19.59^bAB^	20.61^bAB^	22.85^bA^				
	PY	17.91^bB^	20.88^bAB^	21.22^bAB^	22.61^bAB^	23.12^bA^				
	CF-Xa	14.11^bB^	15.88^bAB^	17.73^bAB^	18.82^bA^	19.53^bA^				
	PY-Xa	14.64^bB^	15.41^bB^	16.63^bAB^	17.35^bAB^	19.73^bA^				
Propionic acid (g kg^−1^ DM)	CK	2.65^a^	2.97^a^	3.52^a^	3.59^a^	3.37^a^	0.103	<0.001	0.934	0.561
	Xa	0.93^bB^	0.88^bB^	0.95^bAB^	1.46^bAB^	1.62^bA^				
	CF	1.01^b^	1.22^b^	0.89^b^	1.00^b^	1.07^b^				
	PY	1.25^b^	0.94^b^	1.08^b^	1.01^b^	0.89^b^				
	CF-Xa	0.94^b^	1.02^b^	0.66^b^	0.72^b^	0.63^b^				
	PY-Xa	0.98^b^	0.80^b^	0.82^b^	0.75^b^	0.43^b^				
Butyric acid (g kg^−1^ DM)	CK	1.48^B^	1.71^aAB^	1.54^B^	1.87^aAB^	2.43^aA^	0.070	<0.001	0.654	0.069
	Xa	0.76	0.71^bc^	0.61	1.22^ab^	1.12^ab^				
	CF	0.97	1.03^b^	1.09	0.93^ab^	0.73^b^				
	PY	0.96	1.11^b^	1.19	0.97^ab^	0.77^b^				
	CF-Xa	0.98	0.48^c^	0.32	0.46^ab^	0.42^b^				
	PY-Xa	0.70	0.45^c^	0.61	ND^b^	ND^b^				
Lactic acid/acetic acid	CK	1.50^cA^	1.68^bA^	1.63^bA^	1.77^cA^	0.76^dB^	0.117	<0.001	0.019	0.481
	Xa	3.64^abA^	3.64^aA^	3.32^aA^	2.76^bAB^	2.15^cB^				
	CF	2.97^b^	3.15^a^	3.28^a^	3.15^ab^	3.18^b^				
	PY	3.54^ab^	3.32^a^	3.42^a^	3.29^ab^	3.27^b^				
	CF-Xa	4.36^a^	4.31^a^	4.26^a^	4.42^a^	4.16^a^				
	PY-Xa	4.37^a^	4.34^a^	4.30^a^	4.60^a^	4.35^a^				

a Values with different small letters indicate significant differences among treatments over the same ensiling days, and values with different capital letters show significant differences among ensiling days in the same treatment (*P* < 0.05). *T*, treatment; *D*, ensilage time; *T*×*D*, interaction between treatment and ensilage time; DM, dry matter; FW, fresh material; TN, total nitrogen; CK, control; Xa, only inoculated with combined lactic acid bacteria; CF, only adding consortium CF; PY, adding consortium PY; CF-Xa, adding both consortium CF and Xa; PY-Xa, adding both consortium PY and Xa; ND, not determined.

As the ensiling process progressed, a gradual decrease was observed in the content of all silages (measured by DM). The highest losses were detected in silages treated with CK, followed by those treated with Xa. Because this process was also accompanied by relatively high AA and BA content, the loss could be attributed to heterofermentative fermentation or undesirable bacteria breaking down silage nutrients (8). Another indicator of heterofermentative fermentation is the slow decrease of LA contents and LA/AA in Xa-treated silages from days 30 to 60, which suggests an increased AA production. *Lactobacillus* spp. can metabolize LA to produce AA under sugar-deficient conditions, increasing AA content in ensiling (12). Variable and increasing trends in AA and NH_3_-N content were observed in all silages, and the CF-Xa and PY-Xa treatments resulted in the lowest AA and NH_3_-N contents and the highest LA/AA ratio throughout the ensiling process. Based on the current findings, combining microbial consortia and Xa in treating silages may provide the most benefits to their long-term storage and contribute to a consistent and sustainable supply of high-quality substrates for the production of biofuels.

### Effects of microbial consortia on lignocellulose components of sweet sorghum silages.

The type of treatment and the ensiling duration both had a significant effect on the lignocellulose components (except ADL) of sweet sorghum silages (*P < *0.001) (Table 3). Over the 60 days of ensiling, each of the two consortia, as well as their combination with Xa, were all observed to decrease lignocellulose components of the sweet sorghum silages more than the control and silages exposed to Xa alone. Clearly, synergistic activity between various cellulolytic bacteria promoted the degradation of lignocellulose components. On the other hand, cellulolytic microbes do not just act as specialist cellulose decomposers. Given the complexity of enzymatic systems in many groups, they have the potential to utilize many polysaccharides (28). After 14 days, the contents of neutral detergent fiber (NDF) and acid detergent fiber (ADF) in silages treated with CF-Xa and PY-Xa were both significantly lower contents from silages in the control group or those treated with Xa (*P < *0.05). This corroborates observations in previous studies, which found similar changes in NDF and ADF content when adding lactic acid bacteria and cellulosic enzymes to Leymus chinensis silages (29). Moreover, compared to the control group, silages treated with CF-Xa and PY-Xa also had significantly lower contents of ADL, hemicellulose, and cellulose after 60 days. Across all ensiling processes, cellulose contents were shown to be the lowest in silages treated with PY-Xa. Lactic acid bacteria are known to lack lignocellulolytic enzymes, which degrade structural carbohydrates into WSC (30), whereas consortia CF and PY can convert lignocellulose biomasses into monomeric sugars (hexoses and pentoses), which are directly used for LA fermentation. The hexose and pentoses are then utilized by lactic acid bacteria via the Embden-Meyerhof-Parnas and phosphoketolase pathways, respectively. The presence of lactic acid bacteria also reduces negative product inhibition, which promotes sweet sorghum degradation and fermentation, thereby exhibiting a synergetic effect (31). In other words, lactic acid bacteria assist cellulolytic microbial consortia by promoting downstream utilization, and their synergistic interactions improve the efficiency of bioethanol production.

**TABLE 3:** Influence of microbial consortia on lignocellulosic components in sorghum silage during ensiling^*a*^

Item	Treatment	Data for esiling day:					SEM	*P* value		
		3	7	14	30	60		*T*	*D*	*T*×*D*
NDF (g kg^−1^ DM)	CK	633.30^aA^	625.08^aAB^	609.31^aAB^	600.49^aAB^	597.36^aB^	3.169	<0.001	<0.001	1
	Xa	621.00^abA^	605.60^abAB^	596.23^abAB^	591.99^aAB^	581.41^aB^				
	CF	594.43^abA^	578.62^bcAB^	567.91^bcBC^	560.77^bBC^	552.40^bC^				
	PY	604.47^abA^	577.72^bAcB^	562.41^cB^	558.48^bB^	550.42^bB^				
	CF-Xa	574.96^bA^	565.19^cAB^	553.48^cABC^	540.94^bBC^	533.73^bcC^				
	PY-Xa	570.81^bA^	563.39^cAB^	547.10^cB^	538.47^bBC^	524.23^cC^				
ADF (g kg^−1^ DM)	CK	335.91^a^	333.86^a^	331.98^a^	322.76^a^	315.71^a^	1.870	<0.001	<0.001	1
	Xa	330.44^ab^	329.86^ab^	322.20^ab^	320.52^a^	311.21^a^				
	CF	313.97^bc^	309.32^b^	305.01^ab^	302.34^ab^	294.70^ab^				
	PY	322.31^bcA^	315.47^bAB^	308.74^abBC^	305.34^abBC^	300.37^abC^				
	CF-Xa	311.51^c^	309.58^b^	299.74^b^	289.90^b^	287.11^b^				
	PY-Xa	303.59^cA^	298.30^bAB^	293.67^bAB^	287.34^bAB^	280.78^bB^				
ADL (g kg^−1^ DM)	CK	52.85	51.93^a^	51.57^a^	52.60^a^	51.71^a^	0.557	<0.001	0.409	1
	Xa	52.57	51.72^a^	51.07^a^	50.29^ab^	50.52^a^				
	CF	45.95	44.85^ab^	44.24^ab^	44.58^ab^	43.98^ab^				
	PY	46.35	45.30^ab^	44.57^ab^	44.93^ab^	43.93^ab^				
	CF-Xa	43.40	42.97^b^	41.01^b^	40.89^b^	40.42^b^				
	PY-Xa	43.47	43.13^b^	42.22^ab^	40.76^b^	40.20^b^				
Cellulose (g kg^−1^ DM)	CK	283.06	281.93	280.41^a^	270.16	264.00^a^	1.579	<0.001	<0.001	1
	Xa	277.87	278.14	271.13^ab^	270.23	260.68^a^				
	CF	268.02	264.47	260.76^ab^	257.76	250.73^ab^				
	PY	275.95^a^	270.16^AB^	264.17^abAB^	260.41^AB^	256.44^abB^				
	CF-Xa	268.11	266.61	258.73^ab^	249.01	246.69^b^				
	PY-Xa	260.12	255.17	251.46^b^	246.58	240.58^b^				
Hemicellulose (g kg^−1^ DM)	CK	297.39	291.21^a^	277.34^a^	277.72	281.65^a^	2.057	<0.001	<0.001	1
	Xa	290.55	275.74^ab^	274.03^a^	271.47	270.20^ab^				
	CF	280.46	269.29^bc^	262.90^ab^	258.43	257.69^bc^				
	PY	282.16^a^	262.25^bcB^	253.67^bB^	253.14^B^	250.06^bcB^				
	CF-Xa	263.45	255.61^bc^	253.73^b^	251.05	246.63^c^				
	PY-Xa	267.22	265.08^c^	253.43^b^	251.13	243.45^c^				

a Values with different small letters indicate significant differences among treatments over the same ensiling days, and values with different capital letters show significant differences among ensiling days in the same treatment (*P* < 0.05). *T*, treatment; *D*, ensilage time; *T*×*D*, interaction between treatment and ensilage time; DM, dry matter; FW, fresh material; TN, total nitrogen; CK, control; Xa, only inoculated with combined lactic acid bacteria; CF, only adding consortium CF; PY, adding consortium PY; CF-Xa, adding both consortium CF and Xa; PY-Xa, adding both consortium PY and Xa; NDF, neutral detergent fiber; ADF, acid detergent fiber; ADL, acid detergent lignin.

### Kinetic analysis of the nonstructural carbohydrates of sweet sorghum silages.

**FIG 2:**
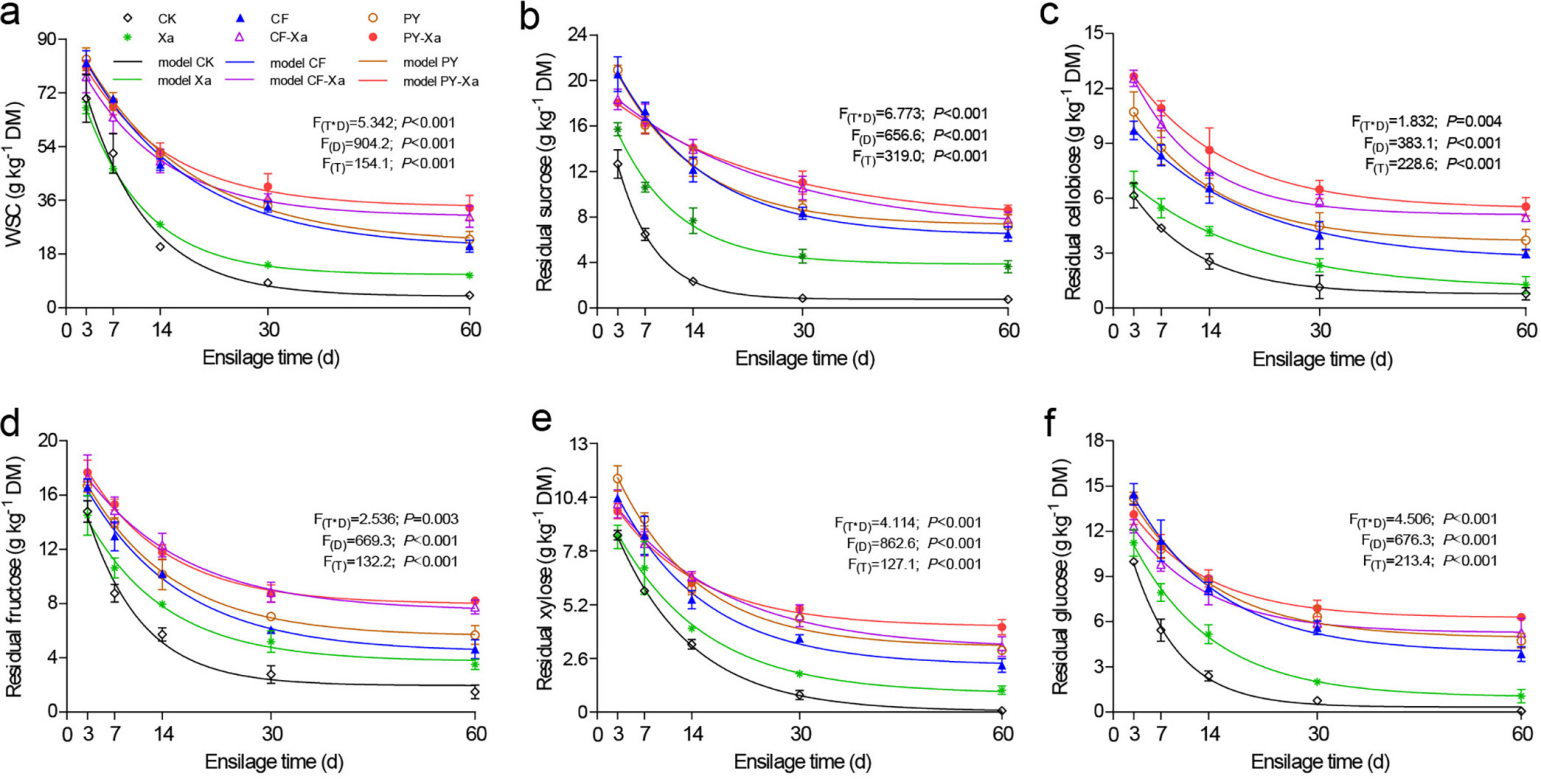
Reduction of water-soluble carbohydrates (a), sucrose (b), cellobiose (c), fructose (d), xylose (e), and glucose (f) in sweet sorghum silages during ensiling. Treatments are as follows: CK, control; Xa, with combined lactic acid bacteria; CF/PY, inoculated with consortium CF/PY, respectively; CF-Xa/PY-Xa, inoculated with consortium CY/PY as well as combined lactic acid bacteria.

Dynamics of the contents of nonstructural carbohydrates during ensiling were quantified to evaluate how microbial consortia affected processes of hydrolysis. A first-order exponential decay model, which strongly fits our data related to the kinetics of nonstructural carbohydrates (*R*^2^ ranging from 0.942 to 0.998), was thus utilized to analyze relevant nonlinear regression dynamics. The type of treatments, duration of ensiling, as well as interaction effects between them all, significantly affect components of nonstructural carbohydrates in sweet sorghum silages (*P < *0.05) (Fig. 2; Table 4). The nonstructural carbohydrate contents in all silages were negatively correlated with the duration of fermentation. After the first 14 days of ensiling, the WSC, sucrose, cellobiose, fructose, glucose, and xylose contents in silages inoculated with CF or PY remained relatively stable through the remaining experimental period, whereas those in CK and Xa silages continued to decline. These trends could likely be attributed to the biodegradation of polysaccharides releasing more sugars than the microorganisms could consume, which is more likely in silages inoculated with only the consortia CF or PY and during later stages of ensiling (32). In contrast, treatments that combine CF or PY with Xa resulted in silages being able to better retain nonstructural carbohydrates. It is important to minimize losses of nonstructural carbohydrates during storage, as they are keys to the biotransformation of biomass into fuels.

**TABLE 4:** Kinetic parameters of nonstructural carbohydrates reduction in sweet sorghum silages based on first-order exponential decay model *y* = *y_m_* + *ae*^−^*^bx^*

Treatment^*a*^	WSC			Sucrose			Cellobiose			Fructose			Xylose			Glucose		
	*y_m_*	*a*	*b*	*y_m_*	*a*	*b*	*y_m_*	*a*	*b*	*y_m_*	*a*	*b*	*y_m_*	*a*	*b*	*y_m_*	*a*	*b*
CK	3.82^d^	94.42^a^	0.110	0.78^c^	20.71^a^	0.184^a^	0.74^c^	7.26^ab^	0.098^a^	1.93^d^	18.36^a^	0.125^a^	0.03^e^	11.08^a^	0.089	0.33^c^	15.05^a^	0.150^a^
Xa	11.11^cd^	77.54^b^	0.111	3.88^bc^	16.44^abc^	0.115^b^	1.00^c^	6.76^b^	0.054^b^	3.76^c^	14.22^ab^	0.096^ab^	0.90^d^	9.88^b^	0.079	0.99^c^	13.08^ab^	0.086^b^
CF	20.01^bc^	75.80^b^	0.064	6.35^a^	18.31^ab^	0.078^bc^	2.58^b^	8.54^ab^	0.057^b^	4.41^bc^	14.86^ab^	0.073^ab^	2.23^c^	10.31^b^	0.076	3.90^b^	13.20^ab^	0.078^b^
PY	21.32^b^	75.11^b^	0.065	7.29^a^	17.48^ab^	0.090^bc^	3.46^b^	9.12^ab^	0.075^ab^	5.58^b^	14.18^ab^	0.078^ab^	3.15^b^	10.59^b^	0.084	4.90^ab^	11.46^bc^	0.081^b^
CF-Xa	29.82^ab^	63.30^bc^	0.093	6.45^a^	13.45^bc^	0.041^c^	5.10^a^	10.06^a^	1.101^ab^	7.44^a^	12.04^b^	0.067^b^	3.02^b^	8.17^c^	0.059	5.23^a^	9.09^bc^	0.090^b^
PY-Xa	33.36^a^	59.20^c^	0.079	7.47^a^	11.89^c^	0.042^c^	5.33^a^	9.38^ab^	0.080^ab^	7.94^a^	12.73^b^	0.084^ab^	4.09^a^	7.20^c^	0.079	6.26^a^	8.92^c^	0.089^b^
SEM	2.400	1.441	0.005	0.588	0.798	0.012	0.444	0.350	0.006	0.521	0.625	0.006	0.341	0.355	0.003	0.543	0.574	0.007
*P* value	<0.001	0.004	0.085	<0.001	0.006	<0.001	<0.001	0.015	0.113	<0.001	0.028	0.053	<0.001	<0.001	0.133	<0.001	<0.001	0.005

a Values in the same column with different superscript letters (a to e) are significantly different (*P* < 0.05). Values with different small letters indicate significant differences among treatments over the same ensiling days, and values with different capital letters show significant differences among ensiling days in the same treatment (*P* < 0.05). *T*, treatment; *D*, ensilage time; *T*×*D*, interaction between treatment and ensilage time; DM, dry matter; FW, fresh material; TN, total nitrogen; CK, control; Xa, only inoculated with combined lactic acid bacteria; CF, only adding consortium CF; PY, adding consortium PY; CF-Xa, adding both consortium CF and Xa; PY-Xa, adding both consortium PY and Xa; NDF, neutral detergent fiber; ADF, acid detergent fiber; ADL, acid detergent lignin.

A lower *a* value or *b* value (except cellobiose) was obtained from all silages inoculated with either microbial consortium, suggesting that the free sugars released during the breaking down of polysaccharides offset consumption by lactic acid bacteria. Treatments also had a significant influence on the *y_m_* values, which represented the theoretical residual nonstructural carbohydrate components after 60 days of ensiling based on the first-order exponential decay model. Compared to the control, the addition of either consortium remarkably improved *y_m_* values. The model indicated that more nonstructural carbohydrates were preserved in CF and PY treatments than the control. Furthermore, CF-Xa and PY-Xa silages were observed to preserve the most nonstructured carbohydrates after ensiling. This corresponds with the high reductions of lignocellulose content in silages subject to these treatments. High nonstructural carbohydrate conservation provides metabolizable energy sources for microorganism-involved biofuel conventions for maximizing biofuel production. Cellulolytic consortia and lactic acid bacteria functioned in a synergistic manner, accelerating the degradation of lignocellulose in the sweet sorghum silage, which would explain the more nonstructural carbohydrates in CF-Xa and PY-Xa silages. Improving steps prior to enzymatic hydrolysis and ethanol production, such as establishing efficient methods of storage, could significantly bolster the effects of pretreatment, increase enzymatic accessibility and availability, and greatly benefit downstream production.

### Morphological changes of sweet sorghum silage.

**FIG 3:**
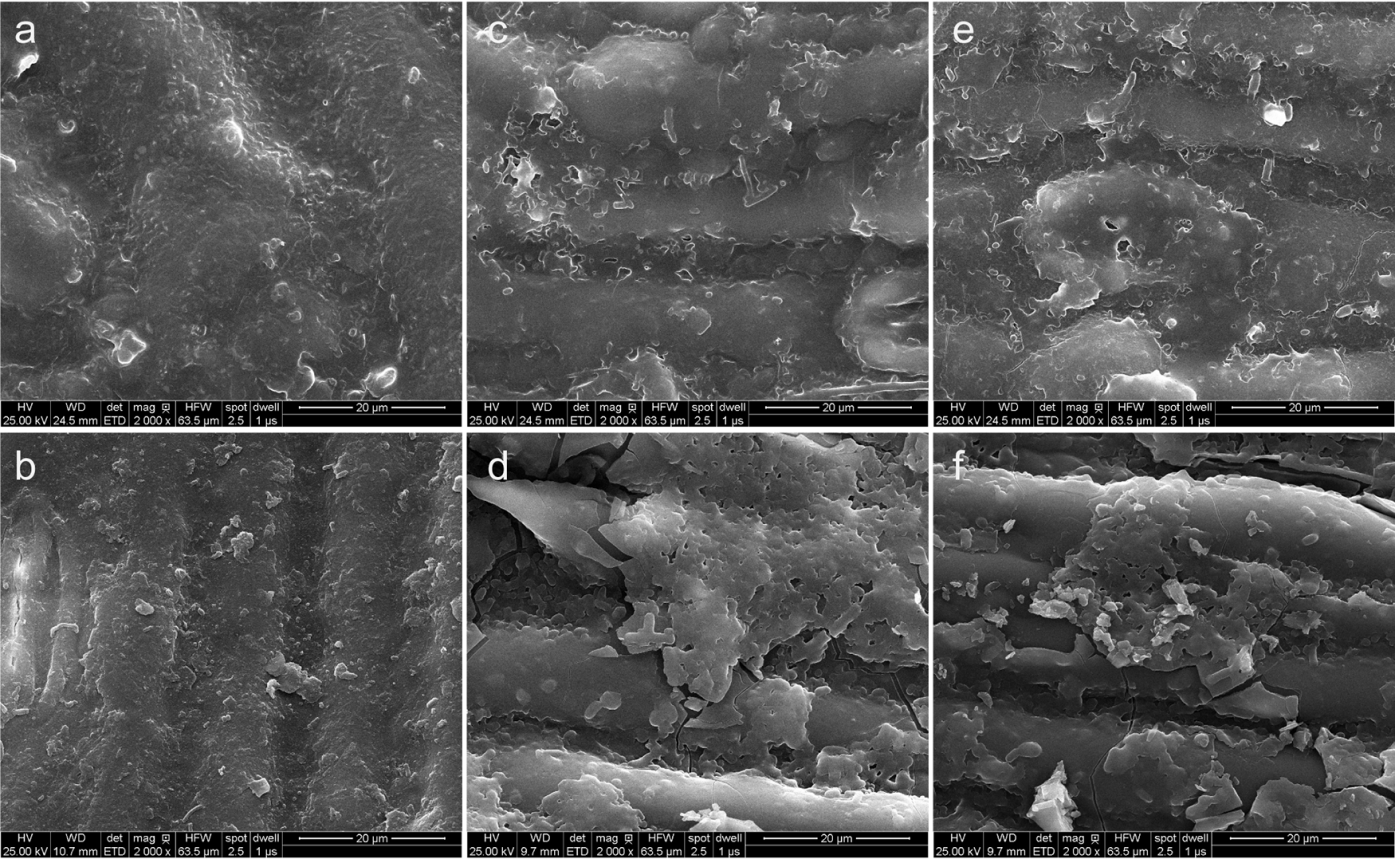
Morphological illustration of the ensiled sweet sorghum over 60 days by electronic microscopy scanning. The image order is as follows: (a) CK, control, no additive; (b) Xa, only inoculated with combined lactic acid bacteria; (c) CF, only adding consortium CF; (d) CF-Xa, adding both consortium CF and Xa; (e) PY, adding consortium PY; (f) PY-Xa, adding both consortiums PY and Xa.

As presented in scanning electron microscope (SEM) images, the surface structure of sweet sorghum ensiled with cellulolytic microbial consortia with or without Xa for 60 days also changed. The surface structures of CK-silages exhibited that changes were smooth, nondecayed, and compact (Fig. 3a), characteristics that would likely hinder enzyme or bacterial attachments. In Xa-treated silages, surface structures were similar, compact, and largely undamaged (Fig. 3b), whereas in sweet sorghum silages inoculated each microbial consortium, CF or PY, we observed many hollowed areas, a sign of partial degradation (Fig. 3c and e). In their review, Liang et al. (33) demonstrated that rumen bacteria attack cut surfaces and lenticels of plants and that they inflict damage to plant cell walls chemically and through adhesion. Treatment with either CF-Xa or PY-Xa caused strong modification and disruptions to the surface structure of sweet sorghum, resulting in fiber detachment, the collapse of cell walls, as well as the formation of pores (Fig. 3d and f). It confirms that, compared to adding microbial consortia or Xa individually, their combined application leads to higher lignocellulolytic degradation potential on sweet sorghum. The degradation of the sweet sorghum surface would form a more accessible area for microbial activity and enzyme hydrolysis, which is, in turn, conducive to downstream processes such as hydrolysis (34).

### Enzymatic hydrolysis and ethanol production from sweet sorghum silages.

**FIG 4:**
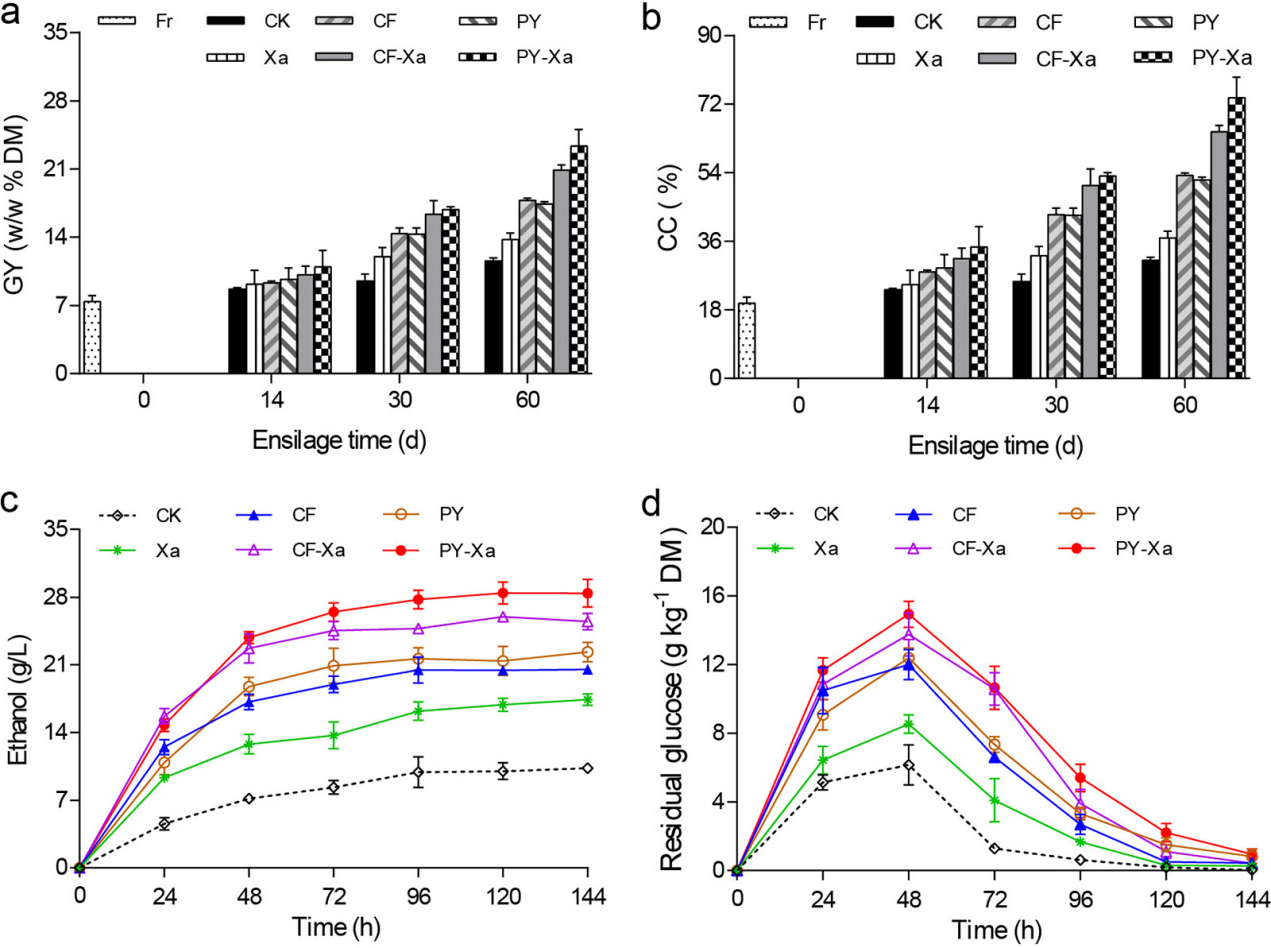
Changes in enzymatic hydrolysis and ethanol production of sweet sorghum silages with different inoculations. (a, b) enzymatic hydrolysis of fresh material and silages (14, 30, and 60 days). (a) glucose yield; (b) cellulose convertibility; (c, d) ethanol yield and residual glucose content when fermenting sweet sorghum ensiled for 60 days. Treatments are as follows: CK, control; Xa, adding combined lactic acid bacteria; CF/PY, adding consortium CF or PY; CF-Xa/PY-Xa, adding consortium CF or PY along with Xa.

Quantifying sugars released from lignocellulose might provide a measure of its accessibility or availability for enzymatic hydrolysis. Thus, enzymatic saccharification reflects how pretreatment affects bioethanol/biogas yields obtained from lignocellulosic biomass. In this work, enzymatic hydrolysis improved notably with ensiling pretreatment using cellulolytic bacteria, leading to higher glucose yield and cellulose convertibility than that observed in fresh materials (Fig. 4a and b). Furthermore, the glucose produced by enzymatic hydrolysis of sweet sorghum silages increased from days 14 to 60 across ensiling. Content of GY and CC also increased in 60-day silages compared to 30-day silages. This indicated that an extended period of ensiling and storage would benefit hydrolysis and degradation, thereby increasing the enzymatic accessibility/convertibility of polysaccharides. Similar outcomes were previously observed in tall fescue, maize, triticale, and giant reed silage (35–37). As expected, the application of cellulolytic microbial consortia remarkably improved carbohydrate availability by enzymatic hydrolysis, evident from significantly higher GY and CC that were detected in CF- and PY-inoculated silages than both control and Xa-treated silages after 30 days. The PY-Xa silage had the highest content of GY (23.34%) and CC (75.37%) on day 60 of ensiling, which was followed closely by CF-Xa (20.90% and 64.76%, respectively), and both were more than 3-fold greater than the results from fresh material. This further demonstrates that inoculation with cellulolytic microbial consortia benefits yields from enzymatic hydrolysis and that a treatment that combines such consortia with Xa represents a highly effective approach for the transformation of cellulose into second-generation bioethanol.

Reducing sugars are key intermediates that bridge cellulolytic processes with downstream bioethanol fermentation (18). Glucose yield reflects cellulose depolymerization in sweet sorghum by enzymatic hydrolyses. When sweet sorghum was inoculated with cellulolytic microbes, rapid saccharification was observed, as demonstrated by the rapidly accumulating glucose levels during the first 2 days, reaching a peak at 48 h (Fig. 4c). These results could be attributable to the rate of glucose production through enzymatic cellulose saccharification being greater than the rate of consumption by the relatively small population of the yeasts during the initial stage of the fermentation. Afterward, residual glucose was rapidly converted into ethanol, resulting in falling glucose concentrations and rising ethanol yields from 48 h until the end of fermentation. This process was enhanced by the inoculation with microbial consortia, and CF and PY treatment groups produced significantly greater ethanol yields than CK and Xa treatments (Fig. 4d). This can be attributed to the diverse, complex microbial strains and their synergistic action to improving enzymatic hydrolysis efficiency. Most prominently, silages treated with PY-Xa had the highest glucose content (14.93 g kg^−1^ DM) at 48 h, as well as the maximum ethanol yield (28.42 g L^−1^), after 120 h of fermentation. The increased bioethanol production was dependent on increased biodegradability with collaboration between cellulolytic microbial consortia and lactic acid bacteria. Our results provide guidelines for suitable methods of the storage and pretreatment of silages and further demonstrate the potential of synergistic microbial consortia in enhancing bioethanol production.

### Conclusions.

Through several indicators of silage quality, including low pH, low NH_3_-N, and high LA/AA ratios, we determined that the ensiling of sweet sorghum and WSC preservation significantly benefited from the addition of synergistic microbial consortia. Synergistic interactions between the cellulolytic microbes and lactic acid bacteria resulted in the alteration of the structure and components of lignocellulose, increased availability of carbohydrates, and improved enzymatic saccharification efficacy, resulting in the enhancement of ethanol production (Fig. S1). The bioaugmented ensiling using these synergistic microbes outlines a cost-effective strategy to store and pretreat sweet sorghum for bioethanol production.

## MATERIALS AND METHODS

### Plants and microbiota.

Materials used in the trial were sourced from experimental fields (containing 5 plots, 35 m^2^ per plot) at Nanjing Agriculture University (Jiangsu, China). The fields were at 32.04°N and 118.88°E, with an altitude of 25 m, and were located in a humid subtropical climate. Sweet sorghum, which was grown for ensiling, was harvested from 3 random plots at the dough stage on 28 August 2020, leaving a stubble of 10 cm. The harvested crops were wilted for 8 h and then cut into segments of 2 to 3 cm. The fresh sweet sorghum contained dry matter (DM) content of 229.26 g per each kg in fresh weight (FW), and its pH was measured to be 5.64. In every kilogram of DM were 90.77 g of water-soluble carbohydrates, 639.92 g of neutral detergent fiber, 331.25 g acid detergent fiber, and 54.11 g acid detergent lignin. Also, the epiphytic aerobic bacteria, yeasts, and lactic acid bacteria were at concentrations of 7.76, 6.53, and 5.81 log CFU g^−1^ FW, respectively.

Two cellulolytic microbial consortia applied in this study, CF (GenBank accession number SAMN16991296) and PY (GenBank accession number SAMN16807469), were both stored at −80°C before the experiment. Following procedures by Li et al. (18), they were cultured in LB media for ~36 to 48 h (optical density at 600 nm [OD_600_] of approximately 0.8) for activation. Dominant genera in CF were *Enterococcu*s (14.9%), Klebsiella (8.5%), Escherichia (8.2%), *Clostridium* (3.46%), *Lactobacillus* (0.52%), Enterobacter (0.11%), *Bacillus* (0.02%), and *Lactococcus* (0.01%) (Table S1). In PY, the most notable genera were Klebsiella (29.8%), *Enterococcus* (19.0%), Escherichia (3.7%), *Lysinibacillus* (0.6%), *Lactobacillus* (0.40%), *Bacteroides* (0.38%), Streptococcus (0.15%), *Cellulosilyticum* (0.08%), and *Bacillus* (0.02%) (Table S1) (18). Consortia CF and PY are known to secrete various carbohydrate-active enzyme (CAZyme) families. In particular, PY holds up to 242 CAZyme-encoding genes, almost twice as many as bacteria from CF (*n* = 141). Most of these genes were glycosyl hydrolases (GHs), largely from the families GH1, GH3, and GH23, and known for their cellulolytic functions. Additionally, the study used a combination of lactic acid bacteria inoculants, specifically, Lactobacillus plantarum, Lactobacillus buchneri, and Pediococcus pentosaceus. To maintain a consistent composition of lactic acid bacteria and ensure the same number of cells were included in each species, individual strains were first prepared individually to the density of OD_0.8_. Then, equal proportions (1.0 mL) of each strain were mixed together.

### Enzyme assays.

The microbial consortia were incubated in a PCS medium adjusted to various pH levels (3.5 to 8.5), and then for 48 h, cultures were kept at 39°C and shaken at 120 rpm. Supernatants from these cultures were centrifuged (10,000 × *g* for 15 min, 4°C) and then used in subsequent enzyme assays. The following substrates were applied in the 3,5-dinitrosalicylic acid method to determine enzyme activity as described by Annamalai et al. (38): microcrystalline cellulose was used to calculate exoglucanase activities, while carboxymethyl cellulose was used for endoglucanase, and salicin was used to calculate xylanase activities (39). In each reaction mixture, 300 μL of the substrate (0.5%, wt/vol) in citrate-phosphate buffer (50 mM, pH 4.8) was mixed with 100 μL of the corresponding crude enzyme (filtered supernatant). Then, the reaction mixture was incubated at 50°C for 30 min. Enzyme activity (U), defined as the amount of enzyme required to release 1 μmol of reducing sugar in 1 min, was evaluated. As well, activity levels of β-glucosidase were measured employing *p*-nitrophenyl glucopyranoside (p-NPG) as a substrate and measuring its release for 30 min in 50 mM citrate-phosphate buffer (pH 4.8) at 50°C (40). Each assay was performed five times.

### Ensilage of sweet sorghum.

Approximately 160 g of chopped sweet sorghum per replicate was treated with the following: (i) CK, control, natural fermentation and distilled water; (ii) CF, consortium CF > 1.5 × 10^6^ CFU g^−1^ FW; (iii) PY, consortium PY > 2.2 × 10^6^ CFU g^−1^ FW; (iv) Xa, the combination of lactic acid bacteria inoculants as described above (active ingredients, L. plantarum* *> 1 × 10^6^ CFU g^−1^ FW, P. pentosaceus* *> 2 × 10^6^ CFU g^−1^ FW, and L. buchneri* *> 1 × 10^6^ CFU g^−1^ FW); (v) CF-Xa, a combination of the CF and Xa treatments; and (vi) PY-Xa, a combination of consortium PY and Xa treatments. Chopped sweet sorghum was manually packed into 150 separate polyethylene bags (320- by 220-mm embossed food saver bags) (5 ensiling days × 6 treatments × 5 replicates = 150) at a density of 0.425 g/cm^3^. Five replicates were created for each combination of 5 ensiling days and 6 treatments, resulting in 150 bags in total, each sealed with the vacuum sealer. These bags were kept at room temperature (~25°C). At days 3, 7, 14, 30, and 60 of ensiling, bags from each treatment were sampled for further analysis.

### Experimental analysis.

On designated dates, five random silos were selected per treatment and opened for analyses. A subsample of each (20 g, FW basis) was extracted in 60 mL of distilled water and then stored at 4°C. After 24 h, extracts were filtered through two layers of cheesecloth as well as a sheet of Whatman filter paper (11 μm), and then their pH values were immediately measured (Hanna pH 211). Subsequently, the extract was used in the calculation of ammonia nitrogen levels (NH_3_-N, following protocol by Broderick and Kang [41]). Ethanol concentrations and contents of organic acids were also analyzed by the Agilent HPLC 1260 (Agilent Technologies, Inc., Berlin, Germany; column, Carbomix H-NP5 [Sepax Technologies, Inc., Santa Clara, CA, USA]; detector, refractive index detector [Agilent Technologies, Inc., Germany]; eluent, 2.5 mmol L^−1^ H_2_SO_4_, 0.5 mL min^−1^; temperature, 55°C).

The remaining fresh sweet sorghum and silage samples were dehydrated in an oven at 65°C to a constant weight and then analyzed to estimate the DM content. Dried samples were finely ground so that they passed through 1-mm screens of a knife mill (model no. FW100) for the analysis of lignocellulose and nonstructural carbohydrate components. Lignocellulose components were evaluated with methods by Van Soest et al. (42), applying heat-stable α-amylase during NDF procedures. The WSC content was determined via a reaction with anthrone reagents followed by colorimetry analysis (43). Mono- and disaccharides were quantified using the Agilent HPLC 1260 (Agilent Technologies, Inc., Waldbronn, Germany; column, Agilent InfinityLab Poroshell 120 HILIC-Z [Sepax Technologies, Inc., Newark, DE, USA]; with the following parameters: temperature: 35°C; eluent A, 0.3% NH_3_·H_2_O; eluent B, acetonitrile; 0.5 mL/min) (18). A scanning electron microscope (SEM) (catalog no. FEI F50; Thermo Scientific, USA) was used to image and analyze surface morphologies of the 60-day silages.

### Fermentation kinetics of the nonstructural carbohydrates.

The first-order exponential decay model was applied to fit data of the nonstructural carbohydrates using GraphPad 6.01 to describe their fermentation kinetics with equation 1 as follows:

(1) $$y=y_{m}+ae^{-\mathrm{bx}}$$

where *y* (g kg^−1^ DM) is the residue at any time *x* (day), *y_m_* (g kg^−1^ DM) is the total residual fraction after 60 days of ensiling, *a* (g kg^−1^ DM) is the consumable fraction, *b* (day^−1^) is the fractional consumption rate of *a*, and *x* is the ensilage time (day).

### Enzymatic hydrolysis.

To assess whether enzyme accessibility had improved, sweet sorghum ensiled for 14, 30, and 60 days were each compared to fresh materials. Commercial enzyme mixtures were prepared as previously described (44) and used for hydrolyzation. Dried and pulverized samples (0.3 g) were homogenized in a citrate-phosphate buffer (0.05 M, pH 4.8) in 100-mL flasks. To prevent microbial contamination, tetracycline (2%, mass/vol) was added. These solutions were kept in an oscillating water bath (50°C, 160 rpm) for 72 h and then heated to 100°C for 10 min to prevent further hydrolysis. The mixtures were then centrifuged (10,000 × *g*, 15 min) and underwent glucose analyses as described above. Glucose yield and cellulose convertibility, both measures of enzymatic hydrolysis efficiency, we calculated using previously described methods (45).

### Ethanol fermentation.

Ethanol production from sweet sorghum silages (60-day) was tested using 100-mL plastic conical flasks using 10% (wt/vol) silage sample powders and a citrate-phosphate buffer. Each flask was loaded with cellulase (65 filter paper unit/g DM) and β-glucosidase (15 cellobiase unit/g cellulose), as well as Saccharomyces cerevisiae (5 × 10^6^ cells g^−1^ DM) for fermentation. The reaction solution was hydrolyzed at 30°C and then kept in a shaking incubator (150 rpm). Samples were taken periodically over 144 h for the determination of ethanol and residual glucose content by high-performance liquid chromatography (HPLC). Saccharomyces cerevisiae CICC1038 (supplied by the CICC) was first precultured in a yeast peptone dextrose medium at 30°C for 36 h, and then collected, centrifuged, washed, and inoculated at an initial cell density of 5 × 10^6^ cells g^−1^ DM.

### Statistical analysis.

All the data were subjected to two-way analysis of variance (ANOVA) with the factors of treatments, ensiling time, and their interactions. Data were modeled using the following general linear models (GLMs):

(2) $$Y_{\mathrm{ij}}=\mu +M_{i}+\;N_{j}+{\left(M\times N\right)}_{\mathrm{ij}}+e_{\mathrm{ijk}}$$

where *Y_ij_* is the dependent variable; μ, the overall mean; *M_i_*, the effect treatments; *N_j_*, the ensiling day; (*M* × *N*)*_ij_*, the interaction effects of treatment and ensiling days; and *e_ijk_*, the residual error. Fermentation kinetics of nonstructural carbohydrates were calculated with ANOVAs. Then, Tukey’s multiple comparisons were applied to compare the differences between treatment pairs.
