# Supplementary material for: Enhancement of Biomass Conservation and Bioethanol Production of Sweet Sorghum Silage by Constructing Synergistic Microbial Consortia
Source: Microbiol Spectr. 2023 Jan 16;11(1):e03659-22. doi: 10.1128/spectrum.03659-22 (PMC9927380; doi:10.1128/spectrum.03659-22)
Supplement: Supplemental file 1 — Supplemental material. Download spectrum.03659-22-s0001.pdf, PDF file, 0.6 MB [file spectrum.03659-22-s0001.pdf]

Supplementary file 1 for

Enhancement of Biomass Conservation and Bioethanol Production of Sweet Sorghum Silage by Constructing Synergistic Microbial Consortia

Yu-Xi Zhu,[a] Xu Zhang,[c] Wen-Chao Yang,[a]* Jun-Feng Li[b]*

[a] College of Plant Protection, Yangzhou University, Yangzhou 225009, China

[b] College of Agro-grassland Science, Nanjing Agricultural University, Nanjing 210095, China

[c] College of Agronomy and Horticulture, Jiangsu Vocational College of Agriculture and Forestry, Nanjing 212400, China

***Corresponding author**:

Wen-Chao Yang, E-mail: wenchaoyn@yzu.edu.cn

Jun-Feng Li, E-mail: lijf@njau.edu.cn

This file includes:

Table S1 to S2

Fig. S1

Supplementary Table

Table S1. The compositions of two microbial consortia CF and PY based on draft genome data.

Classification	Number of sequences (% of assembly)	
	CF	PY
Bacteria	6930533 (96.60%)	6716304 (96.25%)
Firmicutes	1398136 (19.49%)	1454353 (20.84%)
Bacilli	1139560 (15.88%)	1439918 (20.64%)
Lactobacillales	1128119 (15.72%)	1384185 (19.84%)
Enterococcaceae	1074283 (14.97%)	1328275 (19.04%)
Enterococcus	**1071332 (**14.93**%)**	**1324575 (18.98%)**
Lactobacillaceae	40398 (0.56%)	30192 (0.43%)
Lactobacillus	**37471 (0.52%)**	**28041 (0.40%)**
Pediococcus	**-**	**501 (0.01%)**
Streptococcaceae	1672 (0.02%)	11798 (0.17%)
Streptococcus	**938 (0.01%)**	**10539 (0.15%)**
Lactococcus	**613 (0.01%)**	**955 (0.01%)**
Bacillales	3236 (0.05%)	44680 (0.64%)
Bacillaceae	1512 (0.02%)	41675 (0.60%)
Lysinibacillus	-	**40007 (0.57%)**
Bacillus	**1139 (0.02%)**	**1178 (0.02%)**
Clostridia	255554 (3.56%)	11649 (0.17%)
Clostridiales	254988 (3.55%)	11409 (0.16%)
Clostridiaceae	248503 (3.46%)	1755 (0.03%)
Clostridium	**248189 (3.46)**	**1642 (0.02%)**
Enterobacteriaceae	5082409 (70.84%)	4779986 (68.50%)
Klebsiella	**610966 (8.52%)**	**2081094 (29.82%)**
Escherichia	**586846 (8.18%)**	**260598 (3.73%)**
Enterobacter	**7795 (0.11%)**	**1500 (0.02%)**

Note: Information on the compositions of two microbial consortia CF and PY from Li *et al.*, 2021.

Table S2. Summary analysis of the number of CAZyme genes defined in the draft genomes of two microbial consortia CF and PY.

CAZy family	Class name	Number of genes detected		Known activities (http://www.cazy.org.)
		CF	PY	
AA	AA2	1	2	lignin peroxidase, peroxidase, others
	AA3	1	4	cellobiose dehydrogenase, glucose 1-oxidase, others
	AA4	1	0	vanillyl-alcohol oxidase
	AA6	3	7	1,4-benzoquinone reductase
	AA7	1	0	glucooligosaccharide oxidase, chitooligosaccharide oxidase
	AA10	0	2	copper-dependent lytic polysaccharide monooxygenases (LPMOs)
CE	CE1	9	14	acetyl xylan esterase, cinnamoyl esterase, feruloyl esterase, carboxylesterase, others
	CE3	1	1	acetyl xylan esterase
	CE4	2	4	acetyl xylan esterase, others
	CE6	1	0	acetyl xylan esterase
	CE7	1	1	acetyl xylan esterase, cephalosporin-C deacetylase
	CE8	1	1	pectin methylesterase
	CE9	2	2	N-acetylglucosamine 6-phosphate deacetylase, N-acetylgalactosamine-6-phosphate deacetylase
	CE10	5	8	Arylesterase, carboxyl esterase, others
	CE11	1	1	UDP-3-0-acyl N-acetylglucosamine deacetylase
	CE14	1	1	diacetylchitobiose deacetylase, others
GH	GH1	4	12	β-glucosidase, β-galactosidase, β-glycosidase, others
	GH2	2	3	β-galactosidase, β-mannosidase, β-glucuronidase, others
	GH3	2	2	β-glucosidase, 1,4-β-xylosidase, 1,3-β-glucosidase, 1,4-β-glucosidase, others
	GH4	3	10	α-glucosidase; α-galactosidase; α-glucuronidase, others
	GH5	0	1	cellulase, 1,3-β-glucosidase, β-1,4-cellobiosidase, others
	GH8	1	3	cellulase, licheninase, endo-1,4-β-xylanase, others
	GH13	8	11	α-amylase, a-glucosidase, pullulanase, cyclomaltodextrinase, others
	GH15	1	0	glucoamylase, glucodextranase
	GH18	0	3	chitinase, endo-β-N-acetylglucosaminidase
	GH19	0	1	chitinase
	GH23	9	9	lysozyme, peptidoglycan lyase
	GH24	4	2	lysozyme
	GH25	1	0	lysozyme
	GH31	2	3	α-glucosidase, α-1,3-glucosidase, α-xylosidase, others
	GH32	1	2	endo-inulinase, endo-levanase,
	GH36	0	1	α-galactosidase
	GH37	2	3	α-trehalase
	GH38	1	0	α-mannosidase, α-1,3-1,6-mannosidase
	GH39	0	1	β-xylosidase
	GH42	0	2	β-galactosidase
	GH43	0	3	β-xylosidase, β-1,3-xylosidase, xylanase, others
	GH53	0	1	endo-β-1,4-galactanase
	GH63	1	0	α-1,3-glucosidase, α-glucosidase
	GH65	1	1	maltose phosphorylase, trehalose phosphorylase, others

	GH73	2	3	peptidoglycan hydrolase
	GH77	1	2	4-α-glucanotransferase
	GH78	0	1	α-L-rhamnosidase
	GH102	1	1	peptidoglycanlytic transglycosylase
	GH103	1	1	peptidoglycanlytic transglycosylase
	GH104	1	1	peptidoglycanlytic transglycosylase
	GH105	0	2	unsaturated rhamnogalacturonyl hydrolase
	GH108	0	1	N-acetylmuramidase
	GH109	5	14	α-N-acetylgalactosaminidase
	GH114	1	0	endo-α-1,4-polygalactosaminidase
	GH153	0	1	β-1,6-D-glucosamine hydrolase
GT	GT2	10	19	cellulose synthase, chitin synthase
	GT4	10	12	a-glucosyltransferase, lipopolysaccharide N-acetylglucosaminyltransferase
	GT5	1	2	starch glucosyltransferase,starch glucosyltransferase
	GT8	3	4	lipopolysaccharide alpha-1,3-galactosyltransferase
	GT9	4	7	lipopolysaccharide N-acetylglucosaminyltransferase, heptosyltransferase
	GT19	1	2	lipid-A-disaccharide synthase
	GT20	1	1	α-trehalose-phosphate synthase
	GT26	1	3	β-N-acetyl mannosaminuronyltransferase, β-N-acetyl-mannosaminyltransferase, β-1,4-glucosyltransferase
	GT28	1	1	1,2-diacylglycerol 3-β-galactosyltransferase, 1,2-diacylglycerol 3-β-glucosyltransferase, others
	GT30	1	2	α-3-deoxy-D-manno-octulosonic-acid (KDO) transferase
	GT35	2	4	glycogen or starch phosphorylase
	GT51	4	7	murein polymerase
	GT56	1	2	lipid II Fuc4NAc transferase
	GT83	2	1	lipopolysaccharide core alpha-galacturonosyl transferase
PL	PL1	1	0	pectate lyase, exo-pectate lyase, pectin lyase
	PL8	0	1	hyaluronate lyase, chondroitin AC lyase, xanthan lyase
	PL15	0	1	oligo-alginate lyase
	PL22	1	2	oligogalacturonate lyase / oligogalacturonide lyase
CBM	CBM6	0	1	the cellulose-binding function; bind β-1,3-glucan, β-1,3-1,4-glucan, and β-1,4-glucan.
	CBM32	0	1	galactose and lactose-binding module
	CBM34	1	1	granular starch-binding function
	CBM35	0	1	Cellvibrio xylan-degrading enzymes binds module
	CBM41	0	1	α-glucans amylose, amylopectin, pullulan, and oligosaccharide fragments-binding module
	CBM48	3	7	glycogen-binding function
	CBM50	7	8	Binding to chitopentaose
	CBM61	0	1	β-1,4-galactan binding function
	CBM67	0	1	L-rhamnose binding activity

Note: Information on the number of CAZyme genes defined in the draft genomes of two microbial consortia CF and PY from Li *et al.*, 2021.

Supplementary Figure

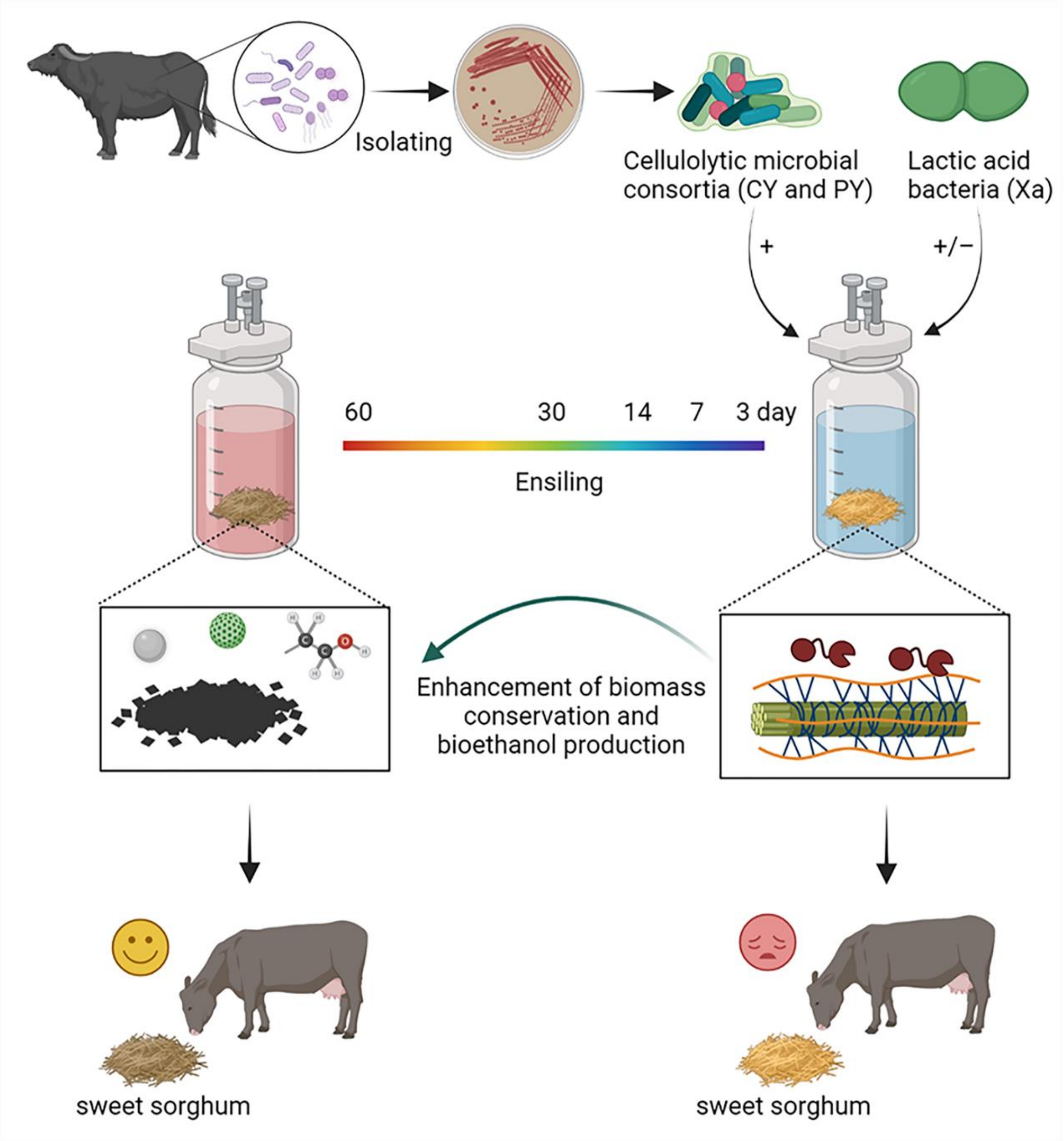

Supplementary Figure S1. A scenario for·enhancement of biomass conservation and bioethanol production of sweet sorghum silage by constructing synergistic microbial consortia.

Reference

Li J, Tang XY, Chen SF, Zhao J, Shao T. 2021. Ensiling pretreatment with two novel microbial consortia enhances bioethanol production in sterile rice straw. *Bioresour Technol* 339: 125507.
